# Genomics of predictive radiation mutagenesis in oilseed rape: modifying seed oil composition

**DOI:** 10.1111/pbi.14220

**Published:** 2023-11-03

**Authors:** Lenka Havlickova, Zhesi He, Madeleine Berger, Lihong Wang, Greta Sandmann, Yen Peng Chew, Guilherme V. Yoshikawa, Guangyuan Lu, Qiong Hu, Surinder S. Banga, Frederic Beaudoin, Ian Bancroft

**Affiliations:** ^1^ Department of Biology University of York York UK; ^2^ Department of Rapeseed Genetics and Breeding, Oil Crops Research Institute CAAS Wuhan China; ^3^ College of Biology and Food Engineering Guangdong University of Petrochemical Technology Maoming China; ^4^ Department of Plant Breeding and Genetics Punjab Agricultural University Ludhiana India; ^5^ Plant Sciences for the Bioeconomy Rothamsted Research Harpenden UK; ^6^ Present address: School of Agriculture, Food and Wine, Waite Research Institute University of Adelaide Glen Osmond SA Australia

**Keywords:** *Brassica napus*, radiation mutagenesis, vegetable oil

## Abstract

Rapeseed is a crop of global importance but there is a need to broaden the genetic diversity available to address breeding objectives. Radiation mutagenesis, supported by genomics, has the potential to supersede genome editing for both gene knockout and copy number increase, but detailed knowledge of the molecular outcomes of radiation treatment is lacking. To address this, we produced a genome re‐sequenced panel of 1133 M_2_ generation rapeseed plants and analysed large‐scale deletions, single nucleotide variants and small insertion–deletion variants affecting gene open reading frames. We show that high radiation doses (2000 Gy) are tolerated, gamma radiation and fast neutron radiation have similar impacts and that segments deleted from the genomes of some plants are inherited as additional copies by their siblings, enabling gene dosage decrease. Of relevance for species with larger genomes, we showed that these large‐scale impacts can also be detected using transcriptome re‐sequencing. To test the utility of the approach for predictive alteration of oil fatty acid composition, we produced lines with both decreased and increased copy numbers of *Bna.FAE1* and confirmed the anticipated impacts on erucic acid content. We detected and tested a 21‐base deletion expected to abolish function of *Bna.FAD2.*A5, for which we confirmed the predicted reduction in seed oil polyunsaturated fatty acid content. Our improved understanding of the molecular effects of radiation mutagenesis will underpin genomics‐led approaches to more efficient introduction of novel genetic variation into the breeding of this crop and provides an exemplar for the predictive improvement of other crops.

## Introduction

Ongoing breeding of new crop varieties with improved resilience and yield is vital for maintaining global food security. Technologies such as the CRISPR‐based genome editing (GE) in plants (Baltes *et al*., 2015; Ji *et al*., 2015; Wang *et al*., 2014) rightly excite the imagination, especially given the range of approaches, such as Associative Transcriptomics (Harper *et al*., 2012), now available to link gene sequence and expression variation with trait variation, thus providing the targets for modification. However, the CRISPR technology has important drawbacks for the commercial deployment required to deliver the promised impact on food security. Varieties of crops meeting food quality standards and adapted to agronomic environments are not always efficiently transformed, freedom to operate is uncertain and whether or not resulting crops should be considered genetically modified (GM) organisms is still contentious in many countries (Altpeter *et al*., 2016; Gao, 2018; Globus and Qimron, 2018). To assure food security, we need also to re‐visit proven ‘traditional’ approaches in combination with the modern genomic approaches.

Mutation breeding has been widely used for crop improvement, with the Food and Agricultural Organization (FAO)/International Atomic Energy Agency (IAEA) Mutant Variety Database (FAO, 2022) listing more than 3200 officially released mutant varieties from 214 different plant species in more than 60 countries. As a ‘traditional’ approach, European regulations do not consider mutation breeding to be a GM technology (Anonymous, 2018). Ionizing radiation has been the most frequently used approach for developing mutant crop varieties (Ahloowalia and Maluszynski, 2001). It carries sufficient energy to displace electrons from atoms or molecules and may comprise particles (e.g. alpha, beta or fast neutrons) or high‐energy electromagnetic waves (such as cosmic, gamma or X‐rays) and is a natural mutagen, being present in the environment (Castillo and Smith, 2017). Artificial ionizing radiation has been used in genetic research as a mutagen since the 1920s (Lewis, 1928a,b; Muller, 1927, 1928) and continues to be used in ‘forward genetics’ studies (*i.e*. the process of identifying lines with altered phenotypes first and finding the gene mutated afterwards) (Campbell *et al*., 2016). PCR‐ and microarray‐based technologies have been used for the identification of deletions (Bruce *et al*., 2009; Li *et al*., 2001; Ríos *et al*., 2008; Rogers *et al*., 2009), but such approaches could not be used as very large populations are needed to be screened. Influential publications describing relatively low viability rates of plants after radiation and purporting a narrow spectrum of mutations (primarily gene‐scale deletions) (Leung *et al*., 2001; Wu *et al*., 2005) led to the approach being largely superseded by chemical mutagenesis for ‘reverse genetics’ studies (*i.e*. the efficient approach of identifying inactivated alleles of genes hypothesized to have a particular function first and confirming the phenotype afterwards). In plants, the only chemical mutagen used extensively is ethyl methanesulfonate (EMS). It selectively alkylates guanine bases with the consequence that during DNA replication a thymine residue replaces a cytosine residue opposite the O‐6‐ethyl guanine formed, which results in a point mutation. Although convenient to use and high mutation loads can be achieved, it induces almost entirely G/C to A/T transitions (Sikora *et al*., 2011; Till *et al*., 2004, 2007). Nevertheless, the Targeting Induced Local Lesions In Genomes (TILLING) technology made the detection of EMS‐induced mutations efficient (McCallum *et al*., 2000) and, following improvements in molecular screens for mutations, has been applied widely in plants (Rigola *et al*., 2009; Wang *et al*., 2012).

Oilseed rape, a crop type of the species *Brassica napus* (AACC, 2*n* = 38), is both a model species for polyploid crops and an important crop in its own right. With worldwide production in 2021/2022 of 71 million metric tons, rapeseed is the world's second largest oilseed crop and the third largest source of vegetable oil according to statistics at http://www.statista.com. Being fellow members of the Brassicaceae (crucifer) family, *Brassica* species are closely related to *Arabidopsis thaliana* and represent an excellent first step for translational biology exploiting fundamental scientific knowledge gained in that species. However, *B. napus* is a recently formed allopolyploid derived by hybridization of *B. rapa* (which contributed the A genome) and *B. oleracea* (which contributed the C genome), each of which themselves possess extensively triplicated genomes indicative of their derivation from a hexaploid ancestor (O'Neill and Bancroft, 2000; Town, 2006; Yang, 2006). Thus, a gene in *A. thaliana* is expected to have multiple orthologues in *B. napus* and each gene in the A genome is likely to have a corresponding gene, which we term a homoeologue, of very similar sequence in the C genome (Cheung *et al*., 2009).

The fatty acid composition of rapeseed oil defines its purpose. Edible oil must contain very little erucic acid, but industrial oil for lubricants and other applications should contain high amounts of erucic acid. Fatty acid biosynthesis has been studied extensively in *A. thaliana*. The major fatty acids in seed oil are derived from the saturated 18‐carbon fatty acid stearic acid. This is largely subjected to desaturation to oleic acid, which can then be elongated to the 22‐carbon erucic acid or further desaturated to form 18‐carbon polyunsaturated fatty acids (PUFAs). The elongation pathway is blocked in rapeseed cultivars grown for edible oil by mutations of orthologues of components of the elongase complex encoded by the *FATTY ACID ELONGASE 1* (*FAE1*) locus (James *et al*., 1995), resulting in rapeseed oil comprising predominantly 18‐carbon fatty acids. Rapeseed cultivars for industrial oil retain the elongation pathway and an increase in erucic acid would be commercially desirable, but its synthesis is limited by dosage of *FAE1* orthologues. The control point in the biosynthesis of PUFAs in *A. thaliana* is the desaturase encoded by the *FATTY ACID DESATURASE 2* (*FAD2*) locus, which catalyses the desaturation of oleic acid to linoleic acid (Miquel and Browse, 1992). Inactivation of *FAD2* orthologues (*Bna.FAD2.A5* and *Bna.FAD2.C5*) in rapeseed results in a reduction in content of polyunsaturated fatty acids (Wells *et al*., 2014), which improves the thermal stability of the oil, particularly in combination with high erucic acid content (Kaur *et al*., 2020).

Despite the consequential genetic redundancy, 21 varieties of rapeseed are listed in the FAO/IAEA Mutant Variety Database (FAO, 2022). However, even with the high mutation rates that can be tolerated by the genomes of polyploid crop species such as oilseed rape, the identification of knock‐out mutants by EMS is inefficient, as only a small proportion (~5%) of the mutations induced lead to protein truncation via the formation of nonsense (stop) codons or splice‐site mutations (Wang *et al*., 2012). For example, only 5 of 102 mutations identified in the JBnaCAB_E oilseed rape EMS TILLING population resulted in the formation of a premature stop codon (Wells *et al*., 2014). Furthermore, tandem gene arrays are common in plant genomes (The Arabidopsis Genome Initiative, 2000), and it is extremely difficult to pyramid knock‐out mutations of very tightly linked genes. Such arrays of genes can, however, be lost by segmental deletions induced by radiation. Also, whereas genome duplications are not induced by chemical mutagens, they are induced by ionizing radiation. Where gene duplication increases the dosage of a gene with additive effects, beneficial impacts on phenotypes may be induced. For example, increasing the copy number of a genome segment in *A. thaliana* containing the locus *FATTY ACID DESATURASE 3* (*FAD3*) increased the content of the omega‐3 PUFA linolenic in seeds (O'Neill *et al*., 2011). Thus, radiation mutagenesis offers scope for induced variation not available via chemical mutagenesis.

The advent of ‘Next Generation Sequencing’ (NGS) enabled the effects of ionizing radiation on plant genomes to be revisited, including studies focussed on *A. thaliana* (Belfield *et al*., 2012). Although this species can tolerate only modest radiation‐induced mutation loads, it was shown that gene‐scale deletions were only a small proportion of the mutations induced by fast neutron irradiation. Point mutations representing a broad range of base changes and small insertion–deletion (InDel) events were much more common; findings reiterated by a similar study in rice (Li *et al*., 2016). Ionizing radiation also induces larger‐scale structural changes in the genome including induction of *de novo* copy number variant (CNV) formation (Arlt *et al*., 2014) as a consequence of incorrect joining of double‐strand breaks by non‐homologous end‐joining, the frequency of which increases greatly in the presence of multiple breaks (Rothkamm and Lobrich, 2002). As well as large‐scale deletions, segmental duplications have previously been observed in the genome of soybean following irradiation (Bolon *et al*., 2014).

The aim of our study was to undertake a comprehensive analysis of the effects of radiation mutagenesis on the genome of a crop species capable of carrying a high mutation load, that is the polyploid crop oilseed rape (*B. napus*). We aimed to: (1) develop and characterize a radiation mutagenesis panel based on genome re‐sequencing. (2) Evaluate the utility of transcriptome re‐sequencing as a lower‐cost approach for large‐scale CNV detection than genome re‐sequencing. (3) Compare the impacts of high‐energy electromagnetic waves (gamma) and particles (fast neutrons). (4) Demonstrate the predictive modulation of traits by both increase and decrease of erucic acid content of seed oil by copy number variation involving large genome segments and by reduction in PUFA content of seed oil caused by a radiation‐induced 21‐base deletion.

## Results

### Development of a genome re‐sequenced radiation mutagenesis panel for oilseed rape

**Figure 1:**
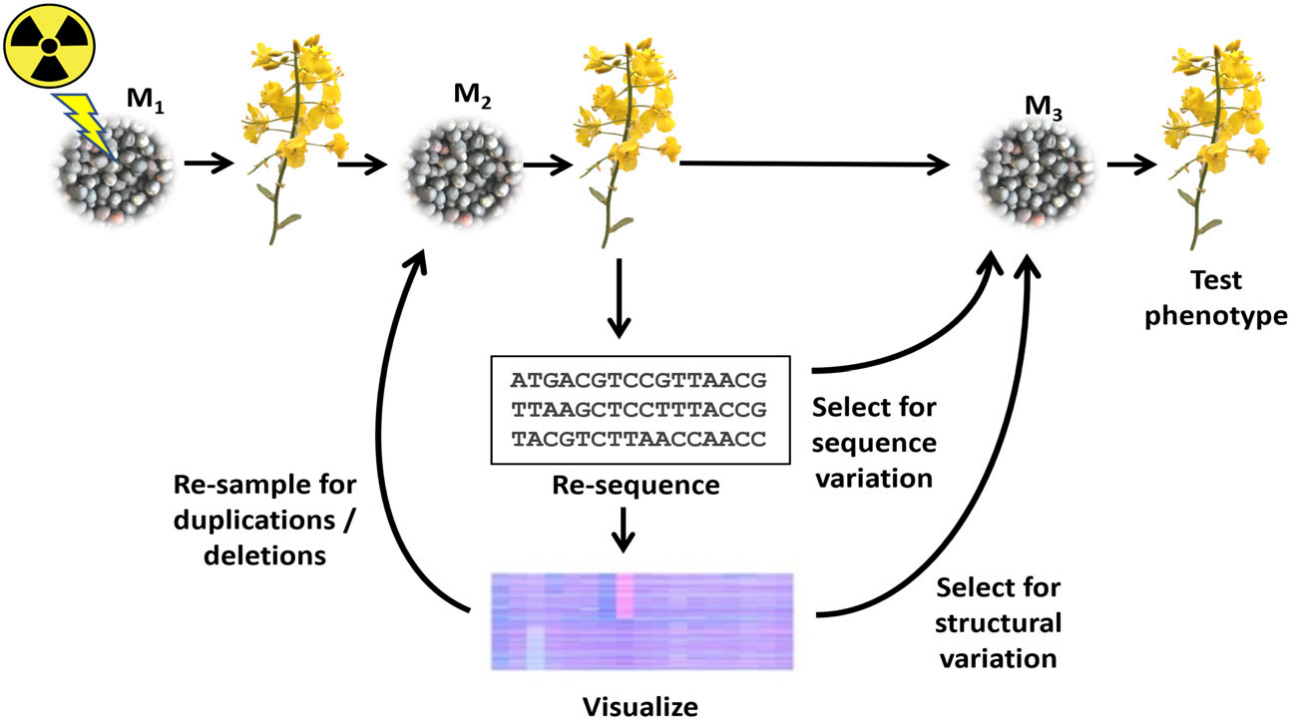
Schematic representation of the development of a genome re‐sequenced radiation panel for reverse genetics.

The main purpose of the genome re‐sequenced radiation mutagenesis panel was to underpin reverse genetic approaches to trait improvement in rapeseed. For population development, we selected a current European winter habit rapeseed variety Maplus. This variety is cultivated to produce industrial oil containing high content of erucic acid and was selected to enable validation by the predictive decrease in erucic acid content in seeds. Seeds (the ‘M_0_’ generation) were treated with a range of doses of either gamma radiation (750–2000 Gy) or fast neutron (FNT) radiation (40–100 Gy) and processed as summarized in Figure 1. Seeds produced by each of the resulting M_1_ generation plants were collected and stored separately. M_2_ seeds were sown (one M_2_ plant produced from each M_1_ plant, apart from a second M_2_ plant being grown from 32 of the M_1_ plants), leaf tissue sampled from the resulting plants for genome re‐sequencing (to 12× coverage), the plants were grown on and bagged for self‐pollination and collection of M_3_ seeds. Both M_2_ and M_3_ generation seeds were stored, with the M_3_ representing the progeny of the genome re‐sequenced plants and the M_2_ representing siblings of the genome re‐sequenced plants. In total, a genome re‐sequenced panel of 1133 M_2_ lines was established.

### Dosage responses of large‐scale copy number variation induced by radiation

**Figure 2:**
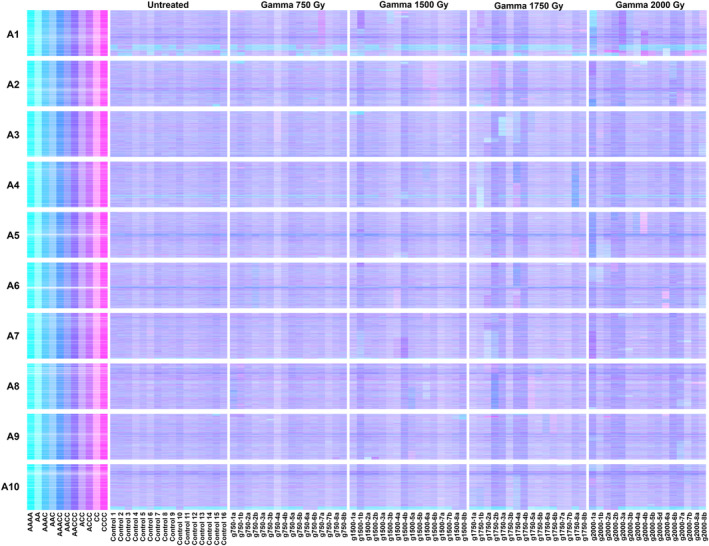
Visualization of genome‐wide copy number variation induced by gamma irradiation, based on genome re‐sequencing. Genome Display Tile Plots were generated based on the relative abundance of genome re‐sequencing reads mapping to genome reference sequences. Quantification is represented in CMYK colour space for homoeologue gene pairs. The cyan component represents abundance of the *Brassica* A genome homoeologue, the magenta component that of the *Brassica* C genome homoeologue. The pairs are plotted in A genome order (chromosomes denoted A1–A10), along with *in silico* combinations to render a diagnostic colour key. The lines analysed are grouped as 16 untreated control plants (Control) and for plants resulting from each of four gamma radiation doses ranging from 750 Gy (g750) to 2000 Gy (g2000), with two sibling progeny (denoted a, b or d) analysed from each of eight irradiated M_1_ plants.

To establish the optimum radiation dose for the focus of genome re‐sequencing, an initial assessment was made of the genomic impact of a range of doses of gamma radiation. Eight pairs of sibling M_2_ plants (i.e. progeny of the same irradiated M_1_ plant) were grown for each of four radiation dosages (750, 1500, 1750 and 2000 Gy), along with 16 untreated plants (to assess any pre‐existing genome structural variation in the seed batch used for irradiation). DNA was prepared and used to produce ~12‐fold genome re‐sequencing data. To assess large‐scale segmental copy number variation, the data were displayed using Genome Display Tile Plots (GDTPs), as shown in Figure 2, which enable detection based upon colour variation that is representative of dosage variation of the homoeologous genomes of *B. napus*. Whole chromosome and segmental copy number variations (the latter being the result of radiation‐induced deletions or duplications) were identified by visual inspection of GDTPs for regions of chromosomes showing skews in colour indicative of variation and are summarized in Table 1. The results show an increase in the frequency of lesions detected as radiation dosage is increased. Comparison between dosages shows little differences in the spectrum of lesion sizes. The frequency of identified deletions from the C genome (mean of 1.52 per plant across all doses) was greater than from the A genome (mean of 0.99 per plant across all doses). Based on our experience of finding structural lesions affecting target genes, we estimate the population size necessary to achieve saturation (meaning an average of 5 instances) of deletion of any given genome region to be ~1000 lines. For duplications, we estimate saturation to require ~4000 lines. Our subjective observation is that M_2_ plants derived from the higher radiation doses showed little by way of clear differences in growth or fertility inhibition compared with plants derived from lower doses, so plants from 1750 and 2000 Gy were used preferentially to build the genome re‐sequenced panel due to their greater mutation load.

**Table 1:** Analysis of genome‐wide copy number variation induced by gamma irradiation

Gamma radiation dosage/Gy	A genome chromosome loss/plant	C genome chromosome loss/plant	A genome chromosome duplication/plant	C genome chromosome duplication/plant	A genome visible deletion/plant	C genome visible deletion/plant	A genome visible duplication/plant	C genome visible duplication/plant	Mean no. deletions and duplications/plant
None	0	0	0	0	0	0	0	0	0
750	0	0	0.06	0	0.5	0.88	0.06	0.20	0.41
1500	0.06	0.06	0	0	0.63	1.13	0.13	0.31	0.55
1750	0.06	0	0.06	0	1.31	1.69	0.56	0.31	0.97
2000	0	0.06	0	0	1.5	2.38	0.63	0.31	1.21

Examination of the GDTPs revealed heterogeneity among the non‐irradiated control lines for homoeologous exchanges. These represent instances of exchange between the genomes of *B. napus*, with the strong cyan bands visible in Figure 2 representing instances of A genome sequences having replaced the corresponding segments of the C genome. This shows that the instability of the *B. napus* genome, in relation to homoeologous exchanges, is sufficient to produce detectable genome structural variation even within breeders' material, which could impact genomic studies.

Comparison of the GDTPs for sibling M_2_ plants (derived from the same M_1_ progenitor) enabled the identification of instances of loss of a genome segment from one plant corresponding to capture of the same genome segment in its sibling. For example, the loss of the upper part of chromosome A5 in line G2000_4b (visible in the plot as a large magenta segment) corresponds to the increased dosage of the corresponding region in its sibling G2000_4a (visible in the plot as a large darker blue segment). In contrast to deletions, the frequency of observed duplications of segments of the C genome (mean of 0.25 per plant across all doses) was lower than of segments of the A genome (mean of 0.38 per plant across all doses).

**Figure 3:**
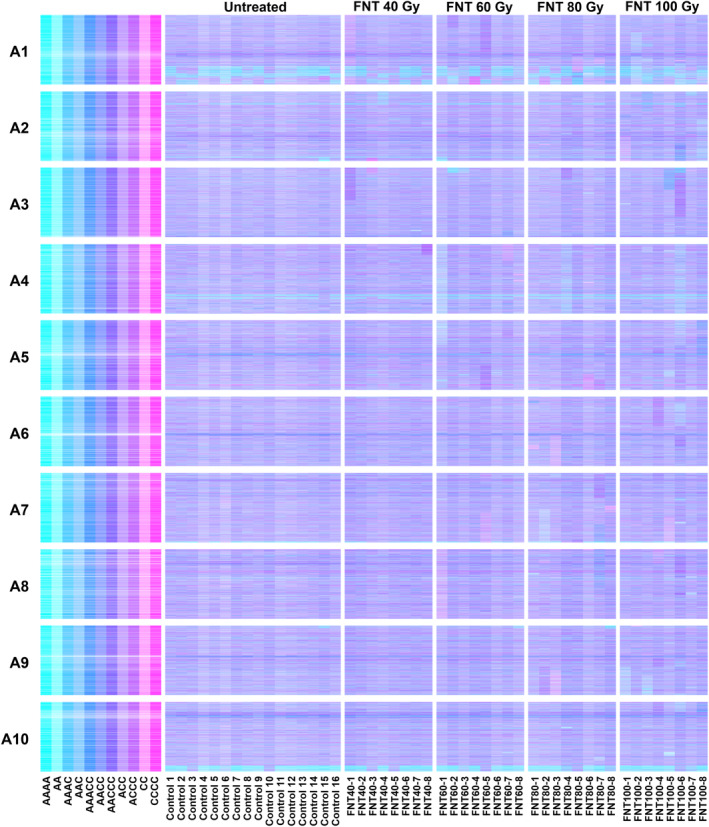
Visualization of genome‐wide copy number variation induced by FNT irradiation based on genome re‐sequencing. Genome Display Tile Plots were generated based on the relative abundance of genome re‐sequencing reads mapping to genome reference sequences. Quantification is represented in CMYK colour space for homoeologue gene pairs. The cyan component represents abundance of the *Brassica* A genome homoeologue, the magenta component that of the *Brassica* C genome homoeologue. The pairs are plotted in A genome order (chromosomes denoted A1–A10), along with *in silico* combinations to render a diagnostic colour key. The lines analysed are grouped as 16 untreated control plants (Control) and for plants resulting from each of four fast neutron radiation doses ranging from 40 Gy (FNT40) to 100 Gy (FNT100), with one progeny analysed from each of eight irradiated M_1_ plants.

To assess any differences in the characteristics of induced genome structural changes induced by different types of radiation, we compared the impacts of four dosages of FNT radiation (40, 60, 80 and 100 Gy) based on genome re‐sequencing, as described for the analysis of gamma irradiated material, for eight M_2_ lines from each dosage. The resulting GDTPs are shown in Figure 3 and whole chromosome and segmental copy number variations are summarized in Table 2. The results show similar numbers of lesions to that observed for the gamma radiation‐derived lines, and similar increasing frequency as radiation dosage is increased. The frequency of identified deletions from the C genome (mean of 1.16 per plant across all doses) was greater than from the A genome (mean of 0.78 per plant across all doses). The frequency of observed duplications of segments of the C genome (mean of 0.53 per plant across all doses) was slightly greater than that of segments of the A genome (mean of 0.44 per plant across all doses). Our subjective observation is that M_2_ plants derived from the higher radiation doses showed little by way of clear differences in growth or fertility inhibition compared with plants derived from lower doses, so plants from 80 and 100 Gy were used preferentially to build the genome re‐sequenced panel due to their greater mutation load.

**Table 2:** Analysis of genome‐wide copy number variation induced by FNT irradiation

Fast neutron radiation dosage/Gy	A genome chromosome loss/plant	C genome chromosome loss/plant	A genome chromosome duplication/plant	C genome chromosome duplication/plant	A genome visible deletion/plant	C genome visible deletion/plant	A genome visible duplication/plant	C genome visible duplication/plant	Mean no. deletions and duplications/plant
40	0.13	0	0	0	0.38	0.25	0	0.25	0.22
60	0	0.13	0	0	0.75	0.63	0.13	0.50	0.50
80	0	0	0	0	1.25	1.38	0.38	0.63	0.91
100	0	0.13	0	0	0.75	2.38	1.25	0.75	1.28

For plants with larger genome sizes than that of *B. napus*, transcriptome re‐sequencing may be a more cost‐effective approach for the detection of radiation‐induced lesions than genome re‐sequencing, as it has been for genome copy number changes arising from homoeologous exchanges (He *et al*., 2017). An analysis of sibling lines following gamma radiation treatment was undertaken based on transcriptome re‐sequencing, as was done for the same lines that were analysed by genome re‐sequencing. Eight pairs of sibling M_2_ plants (i.e. progeny of the same irradiated M_1_ plant) were grown for each of four radiation dosages (750, 1500, 1750 and 2000 Gy), along with 16 untreated plants. The results are shown in Figure S1 and match exactly those produced by genome re‐sequencing, including the inheritance in line G2000_4b of the segment of chromosome A5 lost from line G2000_4a, validating the transcriptome re‐sequencing approach for the analysis of such lesions.

### Impact of copy number variation on erucic acid content of rapeseed

**Figure 4:**
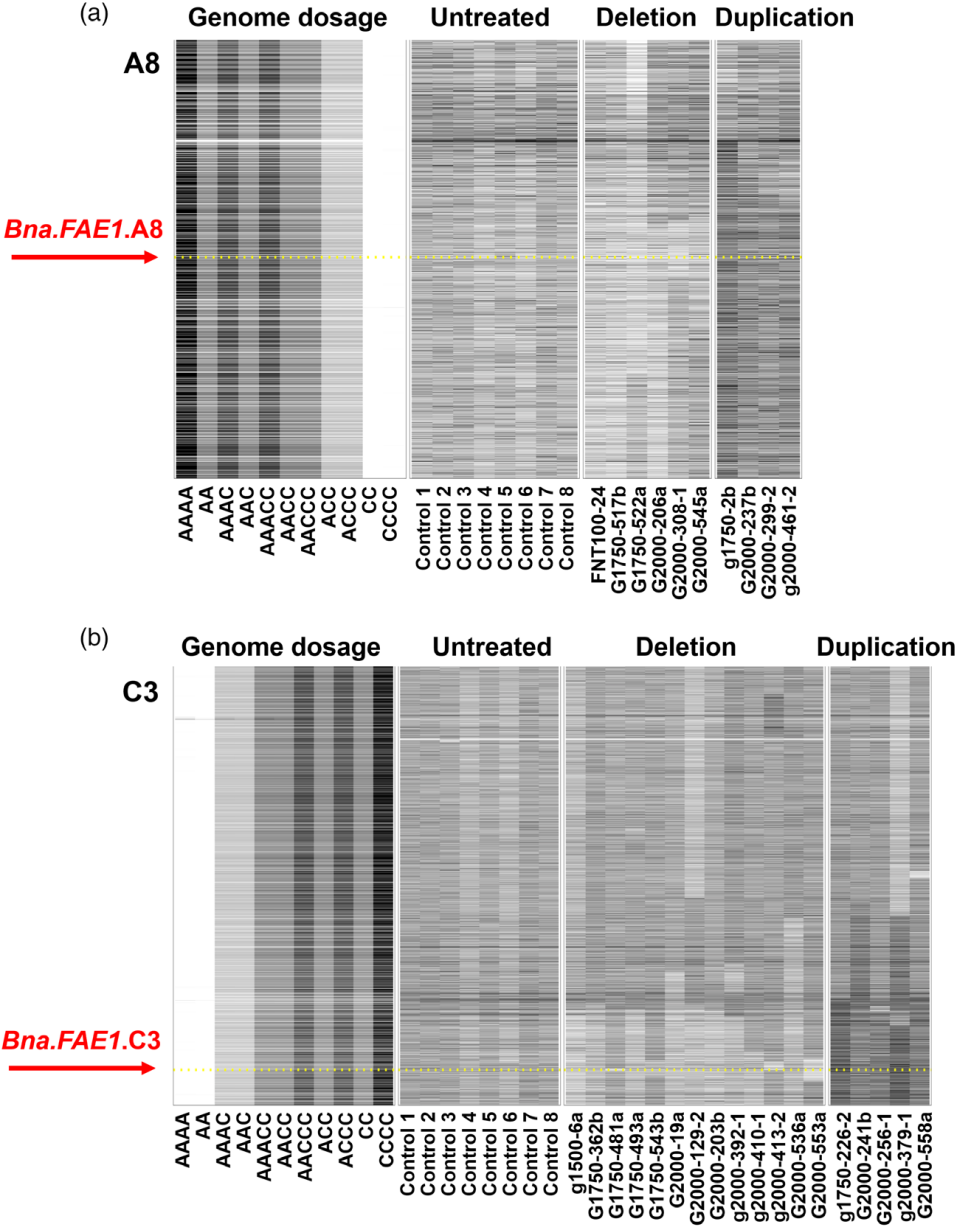
Lines showing genome deletions or duplications affecting the copy number of *FAE1* orthologues. Visualization of genome‐wide copy number variation induced by irradiation, based on genome re‐sequencing. Genome Display Tile Plots were generated based on the relative abundance of genome re‐sequencing reads mapping to genome reference sequences. Lighter tones than balanced genomes (AACC) indicate deletion of genome regions; darker tones than balanced genomes indicate duplication of genome regions. The plots are based on genome order of genes, for the chromosomes on which *FAE1* occur (A8 and C3) and include *in silico* representation of relative copy number.

**Figure 5:**
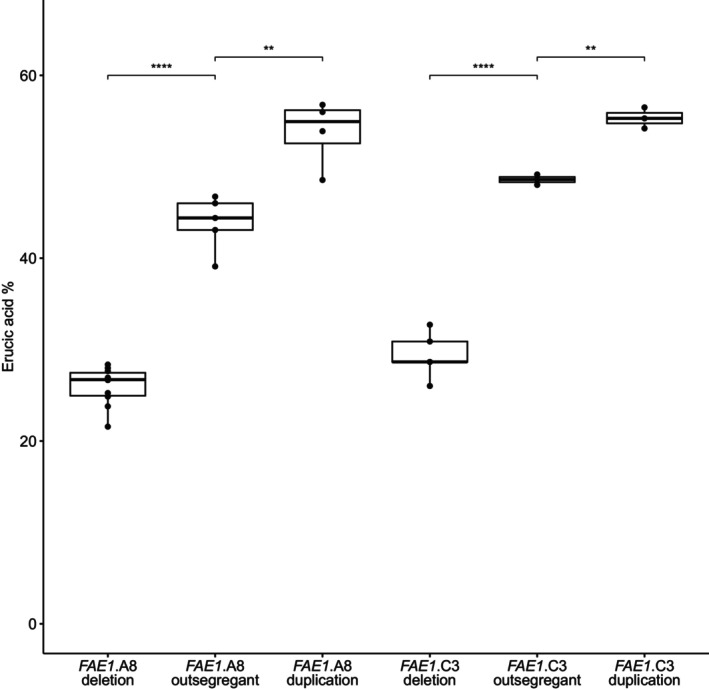
Impact of copy number variation of *FAE1* orthologues on seed oil composition. Content of erucic acid in the seeds of rapeseed lines homozygous for deletion or duplication of *FAE1* orthologues compared with wild‐type outsegregants. *FAE1.A8* deletion in line G2000‐308‐1 (10 plants); *FAE1.A8* outsegregants (5 lines); *FAE1.A8* duplication in line G1750_2b (4 plants); *FAE1.C3* deletion in line G1500_6a (5 plants); *FAE1.C3* outsegregants (3 plants); *FAE1.C3* duplication in line G2000_119a (3 plants). Adjusted *P*‐values for significance level thresholds are: **<0.005; ****<0.00005.

The content of erucic acid in seed oil is an excellent trait for validation of a reverse genetics approach in rapeseed as it is an additive trait, with well‐understood genetics. Associative transcriptomics confirmed the trait to be controlled by two loci with additive effects, in homoeologous positions of chromosomes A8 and C3 (Harper *et al*., 2012; Havlickova *et al*., 2017). These coincided with previously mapped erucic acid quantitative trait loci, *eru1* and *eru2*, for which the control genes are orthologues of the *A. thaliana* gene *FAE1* (Fourmann *et al*., 1998; James *et al*., 1995). We therefore identified the positions of the corresponding two genes (*BnaFAE1.A8* and *BnaFAE1.C3*) in the genome and produced GDTPs for the entire panel to identify large‐scale genome deletions and duplications affecting copies of the genes, as identified by reduced or increased genome representation (i.e. skews in the colour of the GDTPs in the regions containing the target genes). The corresponding CNV lines were re‐plotted for confirmation, as illustrated in Figure 4. In addition, we manually assessed Excel spreadsheets containing raw read depth coverage data for all genes in the reference genome to identify small potential genome deletions and duplications affecting copies of the genes. Four lines were taken forward for trait assessment. Three had large‐scale deletions or duplications and their detection is illustrated in Figure S2: G2000‐308‐1 (~3.0 Mb *BnaFAE1.A8* deletion), G1750_2b (~11 Mb *BnaFAE1.A8* duplication) and G1500_6a (~8.0 Mb *BnaFAE1.C3* deletion). One line has a single gene duplication, and its detection is illustrated in Figure S3: G2000_119a (~1 gene *BnaFAE1.C3* duplication). Seeds (M_3_ generation) were sown, and molecular markers were developed based on single nucleotide polymorphisms between homoeologous positions in the A and C genomes (inter‐homoeologue polymorphisms; IHPs). These are illustrated in Figure S4 and allow the quantitative analysis of copy number of the genomic regions containing *FAE1* orthologues based on the fluorescence signals for the respective bases incorporated during capillary sequencing, providing confirmation of the CNV and enabling its inheritance to be monitored. Homozygous mutant and homozygous control (outsegregant) plants were identified, and their seeds assessed for fatty acid composition. The results are presented in Figure 5 and confirm the expected decrease in erucic acid content (with a corresponding increase in oleic acid content) in the lines from which *FAE1* orthologues have been deleted. The two lines inheriting additional copies of *FAE1* orthologues both showed an increase in erucic acid content, demonstrating the potential to modify traits using radiation mutagenesis to increase gene dosage.

### Variant calling for small‐scale impacts of irradiation

The abundance of large‐scale deletions affecting regions throughout the genome provide an excellent resource for testing multiple knock‐out alleles for predicted gene function. However, chromosome‐scale aberrations may affect genetic stability and reproductive fitness or yield in crops, so commercial exploitation requires loss of function mutants caused by small‐scale events, such as small insertions or deletions (InDels) or single nucleotide variants (SNVs). We therefore screened the panel for such variants using the full‐depth (~12×) genome re‐sequencing data and the Darmor v10 oilseed rape genome reference sequence (Rousseau‐Gueutin *et al*., 2020). Mindful of false positives arising from the numerous steps involved in the production of high‐throughput sequence data (Costello *et al*., 2013), we assessed a range of different types of mutations called within genes of biological interest in rapeseed by PCR amplification and capillary sequencing of the corresponding regions of the genome. The results of these validation tests are provided in Table S1. Of the 26 putative InDels and SNVs tested that had more than two sequence reads of the variant allele, 10 (38%) were validated. We also assessed 57 putative InDels and SNVs that had precisely two sequence reads of the variant allele and these had a much lower validation rate, with only 2 (3.5%) being confirmed. To assess the false‐negative rate, we systematically amplified by PCR and capillary sequenced ~1.8 kb in aggregate of the coding regions of two genes of interest (*FAD2* orthologues on chromosomes A5 and C5) from 770 and 767 lines, respectively. Two mutations were identified, both of which had been detected using the genome resequencing data: one InDel with variant allele read depth = 2 (in line G2000‐137b) and one SNV with variant allele read depth = 4 (in line G1750‐356a). We conclude that the depth of genome resequencing data we produced (~15 Gb per line) is sufficient to detect SNV and InDel variants, but there is a substantial false‐positive rate, particularly in instances where the variant allele read depth is only two. This rate should decrease if greater depth of sequence coverage were to be obtained, but even at the redundancy used in this study, confirmed mutations for dozens of genes could be identified in the panel in a few weeks.

### Predictive reduction in polyunsaturated fatty acid content of rapeseed

Although PUFAs are considered beneficial in edible oil, they reduce thermal stability, limiting industrial applications. Conventional breeding brought about modest reduction in PUFA content but major improvement for both edible and industrial oils was achieved by a focussed reverse genetic approach based on chemical mutagenesis of the *B. napus* chromosome C5 orthologue of *FAD2* (Kaur *et al*., 2020; Wells *et al*., 2014). We aimed to test the utility of radiation‐induced mutagenesis for the production of small mutations suitable for breeding by targeting the *B. napus* chromosome A5 orthologue of *FAD2*. We identified computationally, and confirmed by PCR and capillary sequencing, a 21‐base deletion, resulting in a seven amino acid deletion from the coding region of this gene in line G2000‐137b. Based on our understanding of fatty acid biosynthesis, we predict that mutation of this gene will lead to a reduction on the content of PUFAs, particularly linoleic acid, and an increase in monounsaturated fatty acids, particularly erucic acid.

**Figure 6:**
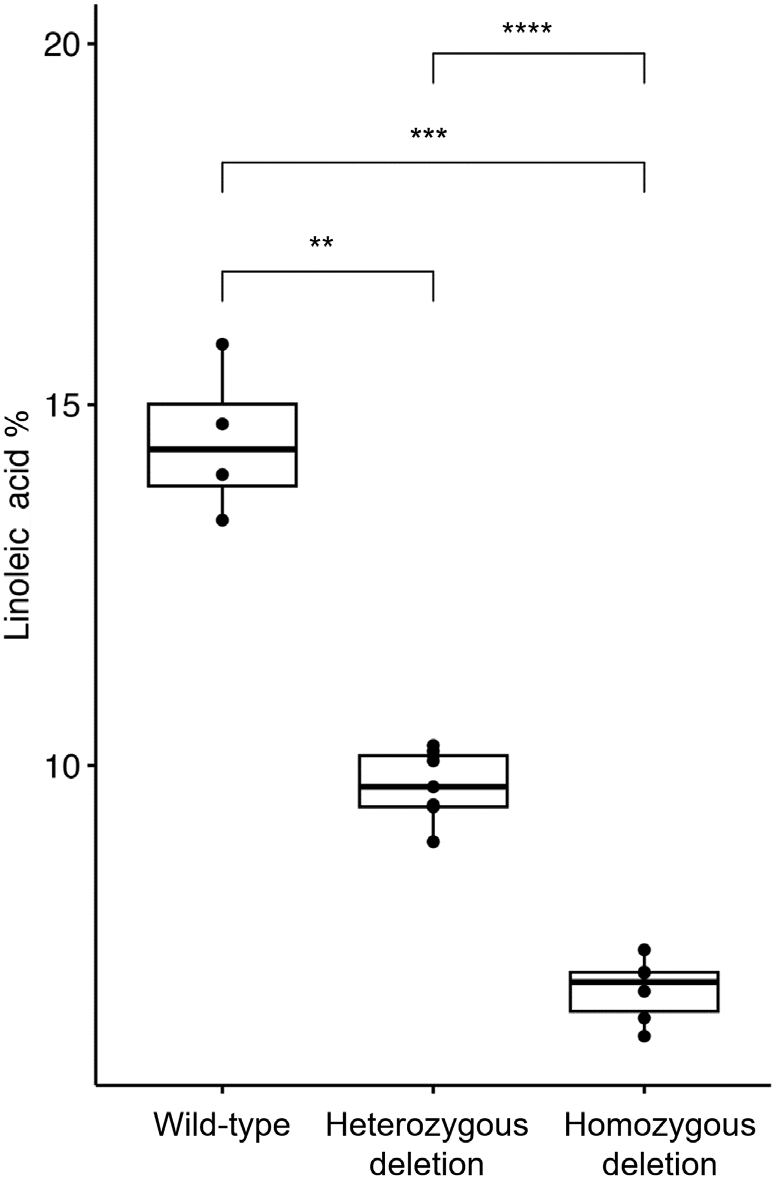
Impact of 21‐bp deletion in *FAD2.A5* gene on linoleic acid content of seed oil. Content of the major polyunsaturated fatty acid in the seeds of rapeseed lines homozygous (6 plants) and heterozygous (9 plants) for the *FAD2.A5* 21‐bp deletion in line G2000‐137b, compared with wild‐type (parental line Maplus) control (4 plants). Adjusted *P*‐values for significance level thresholds are: **<0.005; ****<0.00005; ns, not significant.

Plants from line G2000‐137b (M3 generation) were grown and the genotype confirmed by PCR and capillary sequencing. Both heterozygous and homozygous plants were grown to maturity and seeds collected for fatty acid analysis. Bulk samples of seeds from the plants had fatty acid proportions measured, alongside seeds from the parent variety (Maplus). As shown in Figure 6, the content of PUFAs in the homozygous mutant seeds decreased relative to the wild type (linoleic acid reduced from 15% to 8%) and, as shown in Figure S5, the content of monounsaturated fatty acids increased (oleic acid increased from 15% to 19% and erucic acid increased from 46% to 50%). The predictive manipulation of this trait was therefore confirmed.

## Discussion

Construction of the radiation‐induced mutation panel essentially followed the procedure that we used previously for the development of an EMS‐induced mutation panel for rapeseed (Wells *et al*., 2014), but with the use of radiation treatment of the M_0_ seeds in place of the alkylating agent. The range of radiation dosages used for calibration was designed to exceed that used in previous studies, for example a maximum of 32 Gy FNT for Soybean (Bolon *et al*., 2014) and 350 Gy Gamma for rice (Li *et al*., 2016). Although early growth was slower than that of untreated material, the survival rate of plants subjected to the highest doses was approximately 50%, demonstrating an exceptionally high tolerance of rapeseed to ionizing radiation.

Previous molecular studies of the genome‐wide impact of radiation mainly focussed on the detection of small lesions (SNVs and InDels) by genome resequencing (e.g. Belfield *et al*., 2012; Li *et al*., 2016), but a study using microarray hybridization to estimate genome dosage was effective in identification of both large‐scale deletions and large‐scale duplications (Bolon *et al*., 2014). Sequence‐level variant detection software is well developed and was used to detect SNVs and InDels in our panel. However, our initial aim was to characterize large‐scale structural variants. To do this, we adapted the visualization approach that we developed for the characterization of copy number variation of large genome segments such as those caused by homoeologous exchange or wild relative introgression (He *et al*., 2017, 2021). This approach enabled the visual detection and clear delineation of large‐scale deletion events, with genome re‐sequencing and transcriptome re‐sequencing being equally effective. By re‐sequencing sibling plants derived from the same M_1_ individual, we show for the first time the inheritance in a sibling of a genome segment lost from an irradiated plant. Such duplications are frequent and provide an opportunity to manipulate traits by increasing gene dosage as well as reducing it by deletion.

Our comparisons of lesions induced by Gamma and FNT revealed similar trends of increasing lesion frequency with increasing dosage. More deletions were observed from the C genome than from the A genome, as would be expected given the larger size of the C genome. Inheritance of duplicated C genome fragments was observed at a lower rate than for inheritance of A genome fragments, which is consistent with previous observations of a lower rate of inheritance of C genome segments duplicated by homoeologous exchanges (He *et al*., 2017).

Our genome re‐sequenced radiation mutagenesis panel was produced with the purpose of providing novel germplasm for the predictive improvement of rapeseed crops. We demonstrated this by successfully altering the fatty acid profile of rapeseed oil. First, we confirmed reductions in the erucic acid content of seed oil by deletion of genomic regions containing the long‐chain fatty acid control genes *Bna.FAE1*.A8 and *Bna.FAE1*.C3, providing new alleles to broaden the genetic diversity of the low erucic trait required for edible oil. Second, we increased the erucic acid content of seed oil by duplication of genomic regions containing the same long‐chain fatty acid control, producing the first germplasm with increased content of erucic acid, of use for industrial applications, based on *FAE1* orthologue duplication. Third, we confirmed reduction in PUFA content of seed oil by inactivation of the PUFA control gene *Bna.FAD2*.A5 by a 21‐base deletion, providing a new allele to broaden the genetic diversity of the low PUFA trait, which improves thermal stability of rapeseed oil (Kaur *et al*., 2020).

We have shown that a genome re‐sequenced radiation mutagenesis panel can be a powerful tool for (a) testing hypotheses by copy number variation of target genes within large genomic deletions and duplications, and (b) predictive trait improvement using small‐scale mutations. Thus, we have used genomics to accelerate greatly the ‘traditional’ approach of crop improvement by radiation mutagenesis. Genome re‐sequenced radiation mutagenesis panels could be ‘immortalized’ for use as communal resources by, for example doubled haploid production. The approach supersedes GE for gene knockouts as, once the panel has been established, the identification of targeted knock‐out lines is much more rapid, genome duplications to increase gene dosage are feasible by radiation but difficult by GE and there are currently fewer barriers to commercial deployment. The approach should be applicable to any crop species with a genome sequence available.

## Experimental procedures

### Radiation panel development

Seeds of rapeseed variety ‘Maplus’ (an open‐pollinated European winter habit, low glucosinolate, high erucic acid type) were obtained from their breeder (NPZ Lembke, Hohenlieth‐Hof 1, Germany). Batches were irradiated (as dry seeds) with gamma rays (^60^Co source) at the International Atomic Energy Agency (IAEA), Austria at doses of 750, 1500, 1750 and 2000 Gy, or fast neutrons in the Budapest Research Reactor (BRR) at HAS Centre for Energy Research (AEKI), Hungary at doses of 40, 60, 80 and 100 Gy. Irradiated seeds, defined as the M_1_ generation, were sown in Levington professional F2 compost and grown in long day (16/8 h, 20 °C/14 °C) glasshouse conditions. 4 weeks after sowing, M_1_ plants were vernalised for 10 weeks at 4 °C to induce flowering and then self‐pollinated. M_2_ generation seeds obtained from each M_1_ plant were grown under the same conditions and third true leaves from each of two plant replicates per accession were harvested when they reached ~3 cm in diameter, as close to the mid‐point of the light period as possible. Leaves were harvested separately for processing as individual replicates and immediately frozen in liquid nitrogen. Frozen leaf samples were stored at −80 °C.

### RNA preparation for transcriptome re‐sequencing

Pooled frozen leaf samples were ground in liquid nitrogen. RNA was extracted using the manufacturer's instruction for Omega Biotek EZNA Plant RNA Kit. In total, transcriptome re‐sequencing was completed for 64 *B. napus* M_2_ lines plus 16 control lines that had not been irradiated. Illumina sequencing, quality checking and processing were conducted as described previously (Higgins *et al*., 2012) except that 150 pair end reads obtained from the HiSeq4000 platform were used.

### Transcriptome display tile plots

The sequence reads were mapped to the *Brassica* A and C Pan‐transcriptome (He *et al*., 2021) reference by Burrows‐Wheeler Alignment (BWA) tool (Li and Durbin, 2009) with default parameters, then sorted, indexed and counted by SAMtools (Li *et al*., 2009). The read counts were then normalized by reads per kb per million mapped reads (RPKM) for further analysis. Transcriptome display tile plots (TDTP), which were described in He *et al*. (2017), were generated using the RNA‐seq read counts. The gene pairs used for the plots are provided in Data S1.

### DNA preparation for genome re‐sequencing

DNA was extracted from individual replicate samples. The samples were homogenized in lysis buffer with 3‐mm metal beads using Qiagen (Manchester, UK) TissueLyser II (30/s, 2 min). BioSprint 96 DNA Plant Kit and Qiagen BioSprint 96 Workstation system were used for the DNA extraction. In total, genomes were re‐sequenced for 1133 *B. napus* M_2_ lines plus 16 control lines that had not been irradiated.

### Genome display tile plots

Large structural variants (deletions or duplications) affecting genome copy number variation were detected and visualized by Genome Display Tile Plots (GDTPs). The Illumina HiSeq 6000 platform was used to generate 150‐base paired‐end genomic DNA sequences. The approach to producing the GDTPs is essentially the same as that used for TDTPs described in He *et al*. (2017) except that DNA gene models from the global AC (He *et al*., 2021) are used as the reference sequences and genome sequence reads are mapped. The gene pairs used for the plots are provided in Data S1. The read counts were also normalized as reads per kb per million mapped reads (RPKM) and output as an Excel file for manual analysis of small‐scale structural variants.

### Illustration of read depth coverage for structural variants

The ‘samtools depth’ function in SAMtools (Li *et al*., 2009) was used to compute the read depth for every position along the A08 and C03 chromosomes. Subsequently, the coverage plot was generated using the ‘geom_line()’ function from the ggplot2 package in R.

### Mutation detection by computational analysis

The DNA sequencing reads were mapped to the Darmor V10 reference genome (Rousseau‐Gueutin *et al*., 2020) by Borrows‐Wheeler Alignment tool (v0.7.17) (Li and Durbin, 2009) and indexed by SAMtools (v1.11) (Li *et al*., 2009). Duplicate fragments were marked and eliminated with MarkDuplicates tool in Picard‐Tools (v2.23). SNPs and small InDels (<100 bp) were called by using the HaplotypeCaller tool in GATK (v4.1.3) (McKenna *et al*., 2010). For each sample including controls, a standard filtering criteria (‐‐SnpGap 3, QUAL > 20) was used with BCFtools (v1.10.2) (Li, 2011).

The SNPs and InDels were processed in parallel with the following procedure. First, mutation variants of 16 controls were merged as a master set for filtering. Then, each mutant sample was processed by filtering any mutated position that appeared in the controls using bedtools (v2.29.2) (Quinlan and Hall, 2010). Then, all variants in mutant samples were merged and filtered by occurrence greater than 8. The resultant merged variants (both SNP and InDels) were then processed by restricting target gene region and taken into Ensembl Variant Effect Predictor (VEP) (McLaren *et al*., 2016) for variant effect prediction.

### Mutation validation by targeted Sanger sequencing

Polymerase chain reactions (PCR) were carried out on genomic DNA from lines of the radiation panel. A master mix was made consisting of 10 μL HS Taq.

Mix Red (PCR Biosystems Ltd., UK), 1 μL of each primer (10 μM) and 7 μL of nuclease‐free water, with 1 μL gDNA added to each well. For SNV analysis, the primers 2F (5′ GTGTCTCCTCCCTCCAAA 3′) and 7R (5′ CCTCATAACTTATTGTTGTACCAG 3′) were used for FAD2.A5; for the FAD2_C5 region the primers 1F (5′ GTCTCCTCCCTCCAAAAAGT 3′) and 8R (5′ CAAGACGACCAGAGACAGC 3′) were used (Kaur *et al*., 2020).

The primers used for the validation of other InDels are listed in Table S2. The cycling conditions were 94 °C for 5 min followed by 30 cycles of 94 °C for 30 s, 57 °C for 30 s and 72 °C for 1 min then a final extension of 72 °C for 10 min. PCR products were purified using 1 μL Exonuclease 1 and 1 μL Shrimp Alkaline Phosphatase (SAP) per 10 μL PCR product with an incubation step at 37 °C for 15 min followed by 80 °C for 15 min to terminate the reaction. Samples were sent to Eurofins genomics (Germany) for Sanger sequencing. Sequences were quality trimmed and aligned to a reference sequence using Geneious Prime (Biomatters Ltd., NZ). Differences in the reference were noted, and the find heterozygote function was used to find heterozygous SNPs. All mutations were confirmed by repeating the PCR and sequencing.

### Development of homozygous *FAE1* CNV lines for phenotyping

Lines containing identified copy number variants (M_3_) were self‐pollinated and their progeny genotyped to monitor inheritance of the lesion. The sequences of the A8 and C3 orthologues of *FAE1* differ at only a few nucleotide positions, which are termed inter‐homoeologue polymorphisms (IHPs). Quantitative assays were developed to monitor the relative copy number of the bases at these positions. For these, PCR primers were designed to amplify both gene copies: FAE1 FP (5′ ATGACGTCCGTTAACGTAAAGCTC 3′) and FAE1 RP (5′ CTGACTTACCTGAATCAGAATCAATTTTG 3′). PCR products are capillary sequenced, using primer FAE1 FP as the sequencing primer. The base composition at the IHP positions is analysed quantitatively using software Mutation Surveyor (Softgenetics, State College, PA 16803) to analyse the capillary sequencing chromatograms. IHPs at nucleotide positions 312, 417 and 531 are illustrated in Figure S4.

For the identification of material for phenotyping, DNA was prepared from leaves of individual plants. One young leaf tissue (~1 × 1 cm) per plant was sampled at the 3–4 leaves stage and stored in an Eppendorf tube with a 3 mm metal bead homogenizer at −80 °C until further use. CTAB (cetyl trimethyl ammonium bromide) method was used for the genomic DNA extraction (Murray and Thompson, 1980). Plant tissue was homogenized using QIAGEN's TissueLyser II (2 × 25 frequency/s for 1 min) and 500 μL of 2× CTAB buffer (heated to 65 °C) was added, followed by incubation at 65 °C for an hour. Samples were added with 300 μL of chloroform and isoamyl alcohol solution (ratio 24:1), vortexed and centrifuged at 14 000 ***g*** for 5 min. Approximately 500 μL of the supernatant was transferred to a new tube containing 1 mL of ethanol and sodium acetate solution (ratio 24:1). The tubes were gently inverted to mix, and the samples were incubated in −20 °C freezer for 30 min for DNA precipitation, centrifuged at 14 000 ***g*** for 10 min and supernatant removed. DNA pellets were washed with 500 μL of 70% ethanol and centrifuged for 5 min at 14 000 ***g***. The supernatant was discarded, and the DNA pellet was resuspended in 100 μL of double‐distilled water. DNA concentration was normalized before using it for PCR. PCR for capillary sequencing was performed in 25 μL reaction volumes containing 2 μL sample DNA (~50 ng/μL), 0.5 μL of the forward and reverse primers each (10 μM), 12.5 μL 2× master mix (Thermo Scientific) and 8.5 μL nuclease‐free ddH2O. For amplification, the program used was initial denaturation of 94 °C for 5 min; 35 cycles each with 94 °C for 30 s, 60 °C for 30 s, and 72 °C for 2 min; final extension of 72 °C for 6 min on a Bio‐Rad Tetrad machine. PCR products were purified for sequencing using the Mag‐Bind RNXPure Plus 96‐well equipment and manufacturer's protocol. Purified PCR products were sent to Eurofins Genomics (Ebersberg, Germany; https://www.eurofins.co.uk/) for Sanger sequencing.

### Analysis of seed oil fatty acid composition

Seed oil fatty acid composition was analysed by Gas Chromatography (GC) of Fatty Acid Methyl Esters (FAMEs) using a method adapted from Li *et al*., 2006. Bulk samples (10 randomly selected seeds) of each mutant line and of the Maplus control line were analysed to account for variability in oil content between seeds. Dried seeds were placed in a 3.5 mL glass vial with a PTFE‐lined screw cap and FAMEs were prepared in 1 mL of derivatisation mixture containing 2% (v/v) sulfuric acid, 66% methanol, 28% Toluene as a co‐solvent, 4% 2,2‐dimethoxypropane as a water quencher and 1 mM pentadecanoic acid as an internal standard. Samples were incubated at 85 °C for 4.5 h, after cooling to room temperature 1 mL of 1% NaCl (w/v) was added, and FAMEs were extracted with 2 × 1 mL n‐hexane. Pooled extracts were evaporated under nitrogen and then dissolved in 1 mL of the n‐hexane. The FAME extracts were analysed by GC with a flame ionization detector (FID) on a DB‐23 column (30 m, 0.25 mm i.d., 0.25 μm film; Agilent J&W). The GC conditions were: split mode injection (1:15), injector and flame ionization detector temperature, 240 °C; oven temperature program 150 °C for 1 min, then increasing at 10 °C/min to 240 °C and holding this temperature for 2 min. GC data were normalized using the internal standard and mol% composition generated using response factors for each individual FAME calculated using Supelco 37 Component FAME Mix (Sigma‐Aldrich).

### Statistical analysis of trait data

The R package ‘rstatix’ was used to perform *t* tests to compare the means of two groups of trait (fatty acid content) data. The R package ‘ggpubr’ was used to plot the boxplot with significance levels.

## Accession numbers

All genome and transcriptome re‐sequencing data have been deposited under NCBI BioProject ID: PRJNA945384.

## Author contributions

I.B. conceived and designed the project. L.H., Z.H., L.W., M.B., G.L., F.B., G.S., Y.P.C., G.Y. performed the experiments and analysed the results. Q.H. contributed funding for sequencing. L.H., Z.H., M.B. and I.B. wrote the manuscript, and S.S.B. revised the manuscript.

## Supporting information


**Data S1** Gene pairs for genome and transcriptome display tile plots. Homoeologous gene pairs used for tile plots, including positions in each genome.

**Figure S1** Visualization of genome‐wide copy number variation induced by gamma irradiation, based on transcriptome re‐sequencing. Transcriptome Display Tile Plots were generated based on the relative abundance of mRNAseq reads mapping to CDS gene model reference sequences. Quantification is represented in CMYK colour space for homoeologue gene pairs. The cyan component represents abundance of the *Brassica* A genome homoeologue, the magenta component that of the *Brassica* C genome homoeologue. The pairs are plotted in A genome order (chromosomes denoted A1–A10), along with *in silico* combinations to render a diagnostic colour key. The lines analysed are grouped as 16 untreated control plants (Control) and for plants resulting from each of four gamma radiation doses ranging from 750 Gy (g750) to 2000 Gy (g2000), with two sibling progenies (denoted a, b or d) analysed from each of 8 irradiated M_1_ plants.

**Figure S2** Identification of large‐scale deletion and duplication events in M_3_ radiation‐treated lines. Depth of coverage of genome resequencing reads mapped to chromosomes containing *FAE1* ortholgues. Regions with increased redundancy of coverage (indicating duplication) shown as red bars; regions with reduced redundancy of coverage (indicating deletions) shown as blue bars. The black arrows indicate the positions of the *FAE1* orthologues.

**Figure S3** Identification of a gene‐scale duplication event in an M_3_ radiation line. Excerpt from Excel spreadsheet showing, for genic regions, the depth of coverage of genome sequencing reads mapped to the genome, normalized across the population of radiation lines. Conditional formatting: red (high coverage) to blue (low coverage). Line G2000‐119a shows approximately double representation of the C3 *FAE1* orthologue, indicating a homozygous duplication.

**Figure S4** Inter‐homoeologue polymorphism markers used to identify copy number in plants developed for phenotyping. Excerpts from capillary sequencing chromatograms in regions of polymorphisms between A and C genome orthologues of *FAE1* following co‐amplification of both copies by PCR. The black arrows indicate the position of the polymorphism. The base indicative of each genome is indicated above the chromatograms, for each of three IHPs (IHP312, IHP417 and IHP531).

**Figure S5** Impact of 21‐bp deletion in *FAD2.A5* gene on oleic and erucic acid content of seed oil. Content of the major mono‐unsaturated fatty acids in the seeds of rapeseed lines homozygous and heterozygous for the *FAD2.A5* 21‐bp deletion compared with wild‐type (parental line Maplus) control. Adjusted *P*‐values for significance level thresholds are: **<0.005; ****<0.00005; ns = not significant.

**Table S1** Validation of InDels and SNVs. Reference and variant allele statistics from validation analyses.

**Table S2** Primers used in the validation of InDels. Oligonucleotide primers used for validation analyses.

## Data Availability

The data that support the findings of this study are available from the corresponding author upon reasonable request.
