# Supplementary figures and images for: Genomics of predictive radiation mutagenesis in oilseed rape: modifying seed oil composition
Source: Plant Biotechnol J. 2023 Nov 3;22(3):738–50. doi: 10.1111/pbi.14220 (PMC10893948; doi:10.1111/pbi.14220)

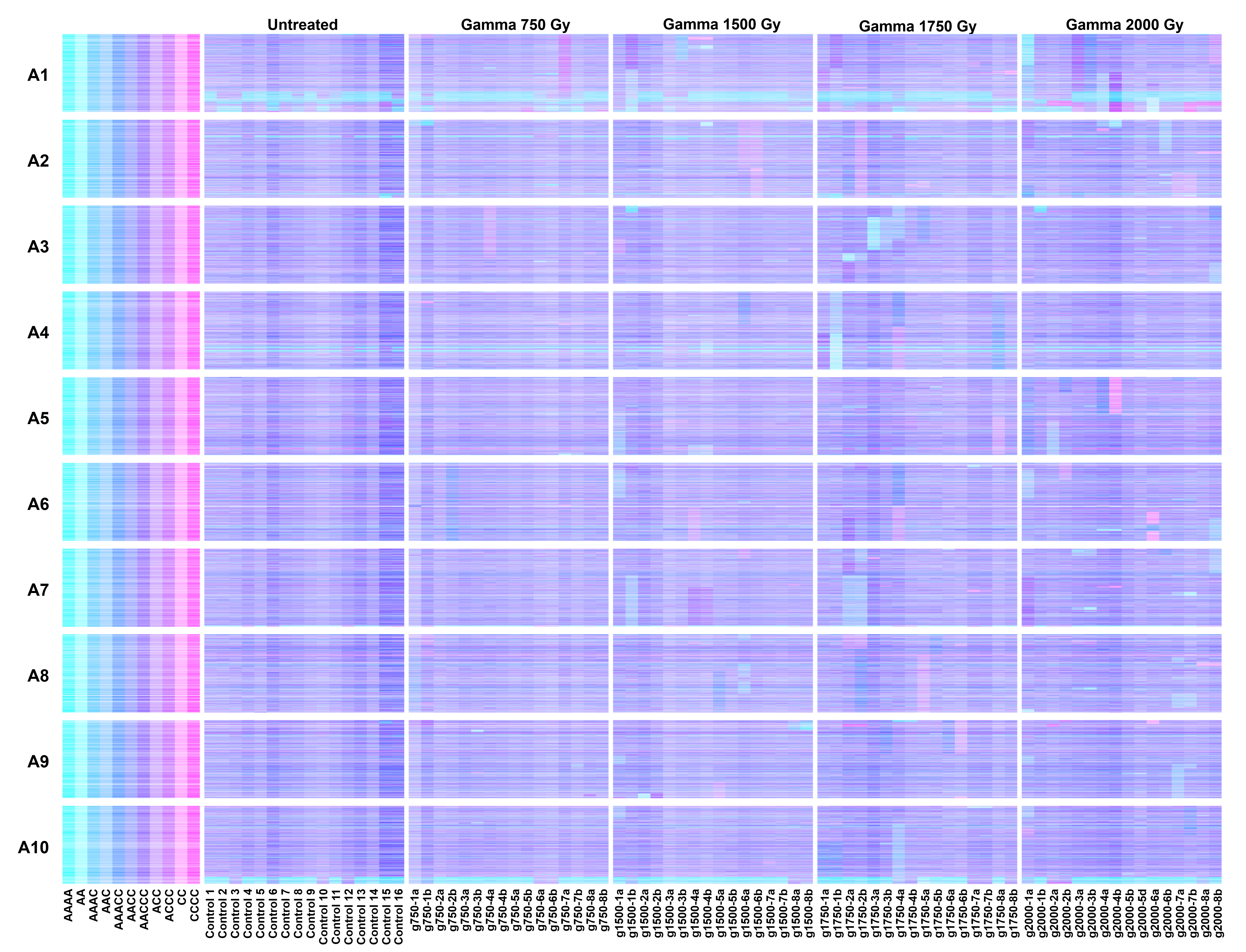

Supplement: Supplementary file 2 — Figure S1 Visualization of genome‐wide copy number variation induced by gamma irradiation, based on transcriptome re‐sequencing. Transcriptome Display Tile Plots were generated based on the relative abundance of mRNAseq reads mapping to CDS gene model reference sequences. Quantification is represented in CMYK colour space for homoeologue gene pairs. The cyan component represents abundance of the Brassica A genome homoeologue, the magenta component that of the Brassica C genome homoeologue. The pairs are plotted in A genome order (chromosomes denoted A1–A10), along with in silico combinations to render a diagnostic colour key. The lines analysed are grouped as 16 untreated control plants (Control) and for plants resulting from each of four gamma radiation doses ranging from 750 Gy (g750) to 2000 Gy (g2000), with two sibling progenies (denoted a, b or d) analysed from each of 8 irradiated M1 plants. [file PBI-22-738-s003.png]

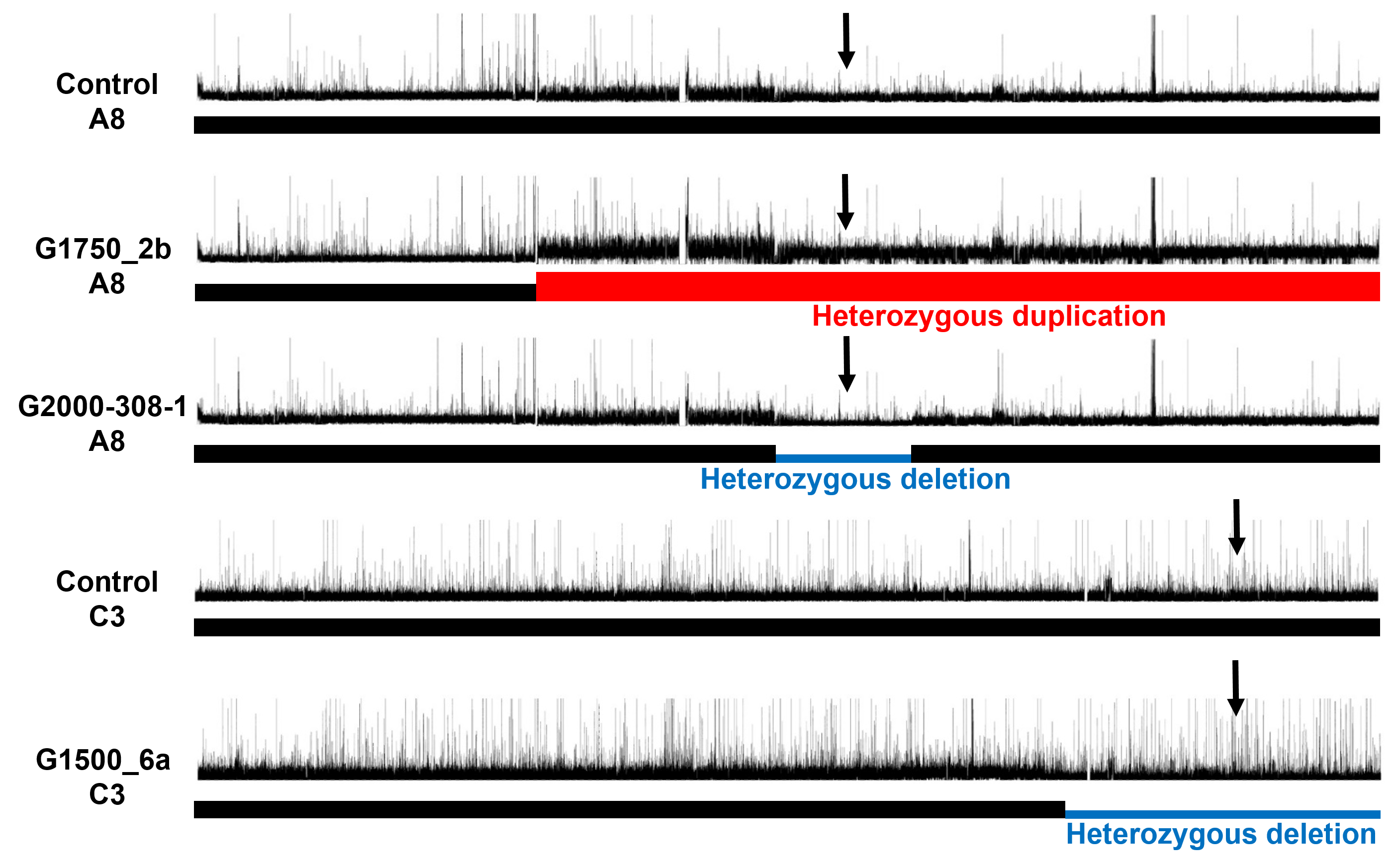

Supplement: Supplementary file 3 — Figure S2 Identification of large‐scale deletion and duplication events in M3 radiation‐treated lines. Depth of coverage of genome resequencing reads mapped to chromosomes containing FAE1 ortholgues. Regions with increased redundancy of coverage (indicating duplication) shown as red bars; regions with reduced redundancy of coverage (indicating deletions) shown as blue bars. The black arrows indicate the positions of the FAE1 orthologues. [file PBI-22-738-s001.png]

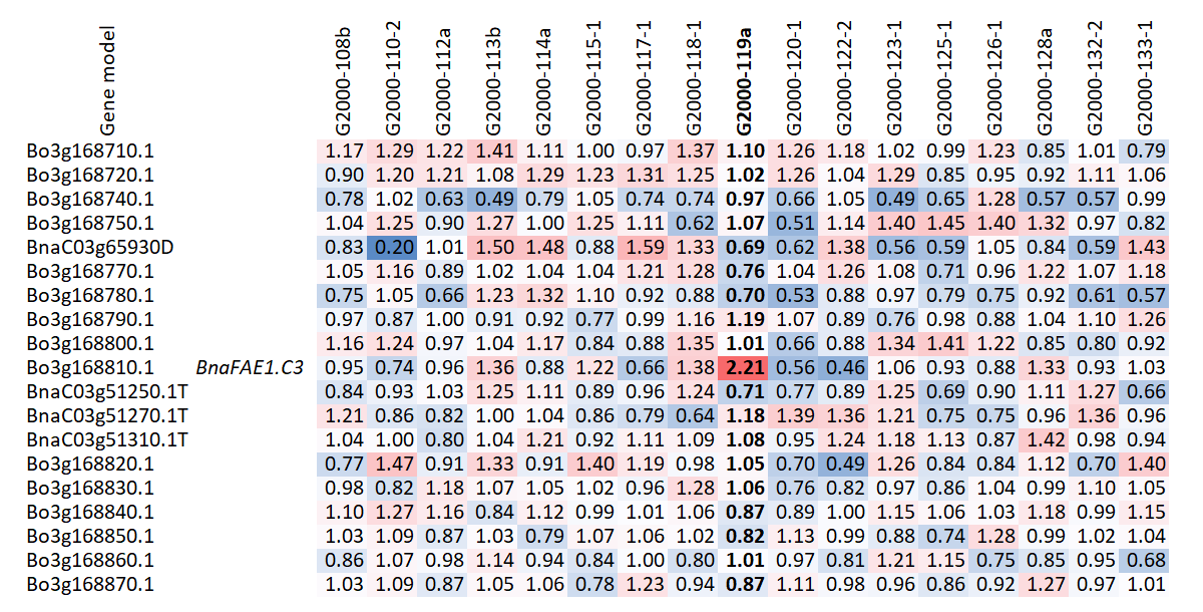

Supplement: Supplementary file 4 — Figure S3 Identification of a gene‐scale duplication event in an M3 radiation line. Excerpt from Excel spreadsheet showing, for genic regions, the depth of coverage of genome sequencing reads mapped to the genome, normalized across the population of radiation lines. Conditional formatting: red (high coverage) to blue (low coverage). Line G2000‐119a shows approximately double representation of the C3 FAE1 orthologue, indicating a homozygous duplication. [file PBI-22-738-s005.png]

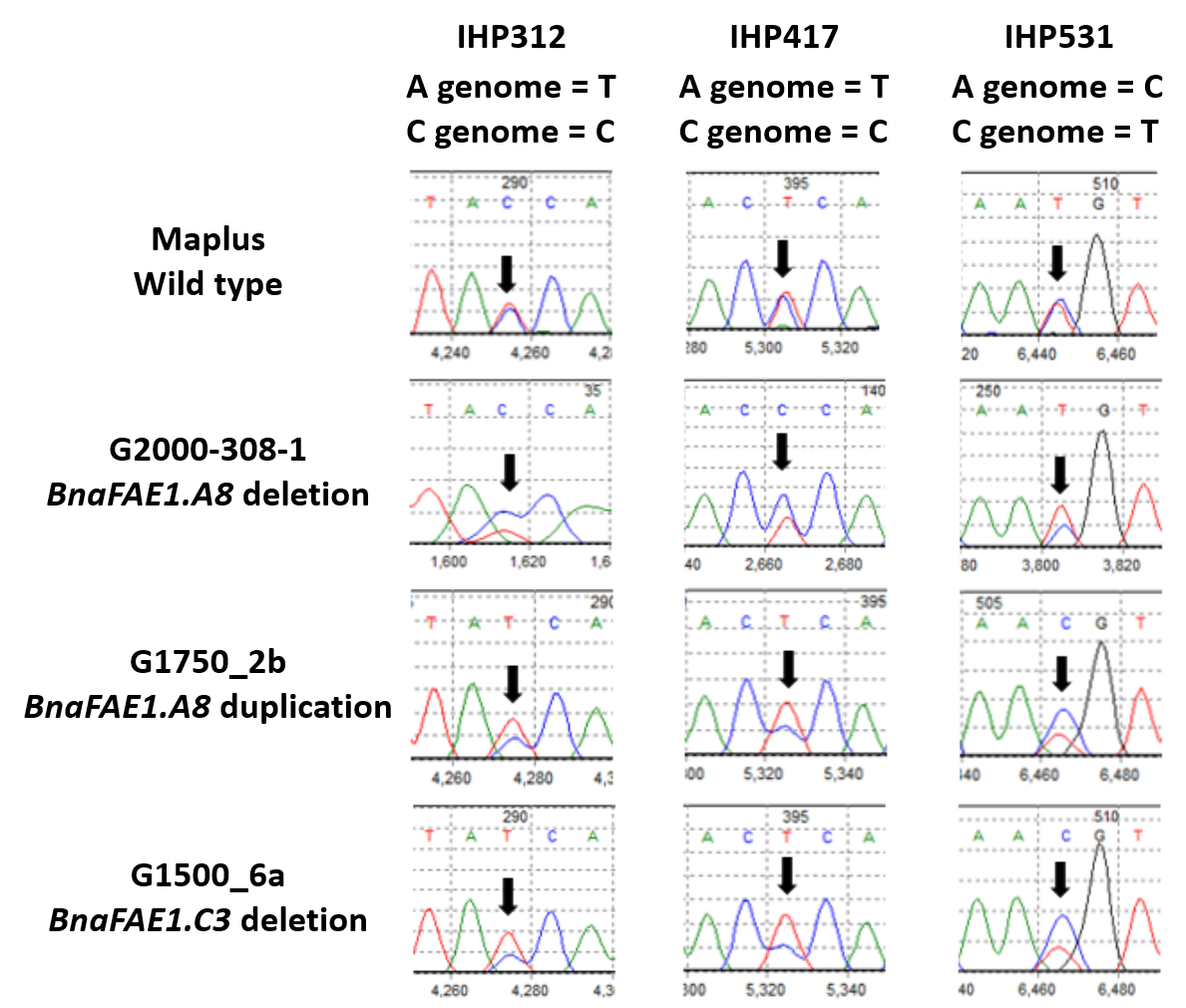

Supplement: Supplementary file 5 — Figure S4 Inter‐homoeologue polymorphism markers used to identify copy number in plants developed for phenotyping. Excerpts from capillary sequencing chromatograms in regions of polymorphisms between A and C genome orthologues of FAE1 following co‐amplification of both copies by PCR. The black arrows indicate the position of the polymorphism. The base indicative of each genome is indicated above the chromatograms, for each of three IHPs (IHP312, IHP417 and IHP531). [file PBI-22-738-s006.png]

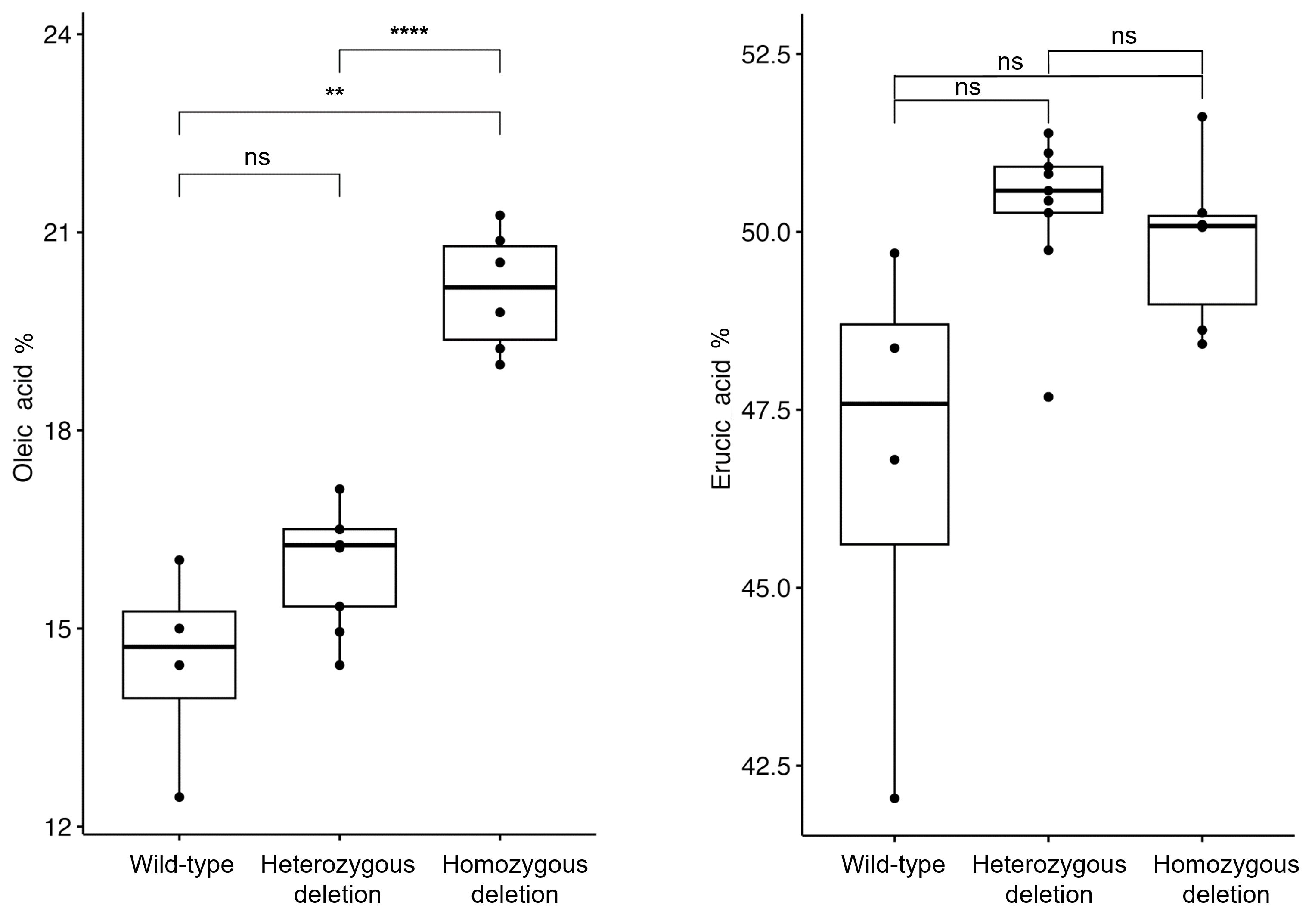

Supplement: Supplementary file 6 — Figure S5 Impact of 21‐bp deletion in FAD2.A5 gene on oleic and erucic acid content of seed oil. Content of the major mono‐unsaturated fatty acids in the seeds of rapeseed lines homozygous and heterozygous for the FAD2.A5 21‐bp deletion compared with wild‐type (parental line Maplus) control. Adjusted P‐values for significance level thresholds are: **<0.005; ****<0.00005; ns = not significant. [file PBI-22-738-s008.png]
