# Supplementary material for: Genomics of predictive radiation mutagenesis in oilseed rape: modifying seed oil composition
Source: Plant Biotechnol J. 2023 Nov 3;22(3):738–50. doi: 10.1111/pbi.14220 (PMC10893948; doi:10.1111/pbi.14220)
Supplement: Supplementary file 7 — Table S1 Validation of InDels and SNVs. Reference and variant allele statistics from validation analyses. [file PBI-22-738-s002.docx]

Supplementary Information File 2. Validation of InDels and SNVs.

| **Target gene** | **Line** | **Nucleotide position** | **Reference allele** | **Read depth reference allele** | **Variant allele** | **Read depth variant allele** | **Validated?** |
| --- | --- | --- | --- | --- | --- | --- | --- |
| *Bna.GTR1.A1* | G2000-564a | A01:14825213 | TA | 8 | T | 2 | No |
| *Bna.GTR1.A1* | G2000-265-1 | A01:14826749 | GT | 11 | G | 2 | No |
| *Bna.GTR1.A1* | FNT60_1a | A01:14827554 | CA | 9 | C | 2 | No |
| *Bna.CER4-like.A1* | g2000-347-2 | A01:2085586 | A | 10 | AGCTCCGTGT | 2 | No |
| *Bna.CER4-like.A1* | G2000-236a | A01:2085809 | GA | 6 | G | 2 | No |
| *Bna.VTE4.A2b* | G1750-328a | A02:16358262 | TC | 12 | T | 2 | No |
| *Bna.VTE4.A2a* | G2000-101b | A02:8953237 | CCGT | 11 | C | 2 | No |
| *Bna.FAD3.A3* | G2000-130-1 | A03:7233038 | CA | 10 | C | 2 | No |
| *Bna.FAD3.A4* | G2000-104b | A04:16657399 | AC | 6 | A | 2 | No |
| *Bna.FAD3.A4* | G2000-145b | A04:16659808 | CCTTT | 6 | C | 6 | Yes |
| *Bna.eIF(iso)4E.A4* | G1750-512a | A04:7767723 | CTA | 1 |  | 2 | No |
| *Bna.eIF(iso)4E.A4* | G1750-430a | A04:7768449 | CATTG | 11 | C | 2 | No |
| *Bna.eIF(iso)4E.A4* | G2000-119-1 | A04:7768451 | GGAGAGGTAGAAGAT | 0 | G | 3 | Yes |
| *Bna.FAD2.A5* | g2000-405-1 | A05:36891562 | TAG | 8 | T | 2 | No |
| *Bna.FAD2.A5* | G2000-137b | A05:36892995 | CCACCACTTACTTCCCTCTCCT | 11 | C | 2 | Yes |
| *Bna.FAD2*.A5 | FNT100-24 | A05:36893503 | T | 7 | C | 2 | No |
| *Bna.FAD2*.A5 | G2000-226b | A05:36893534 | G | 11 | T | 2 | No |
| *Bna.FAD2*.A5 | G2000-184a | A05:36893565 | T | 6 | G | 2 | No |
| *Bna.FAD2*.A5 | G1750-542a | A05:36893665 | A | 7 | G | 2 | No |
| *Bna.FAD2*.A5 | g2000-323-1 | A05:36893692 | C | 10 | T | 2 | No |
| *Bna.FAD2*.A5 | G2000-191a | A05:36893697 | T | 2 | G | 3 | No |
| *Bna.FAD2*.A5 | g2000-496-2 | A05:36893724 | C | 7 | T | 2 | No |
| *Bna.CytP450.A5* | G1750-442a | A05:5735885 | CCT | 15 | C | 9 | No |
| *Bna.CytP450.A5* | G2000-127-1 | A05:5736295 | C | 8 | CATTCTCCAGCGAGTCTAG | 2 | No |
| *Bna.CytP450.A5* | G2000-328-2 | A05:5736295 | C | 7 | CATTCTCCAGCGAGTCTAG | 2 | No |
| *Bna.CytP450.A5* | g2000-454-1 | A05:5736436 | CT | 10 | C | 2 | No |
| *Bna.CytP450.A5* | G2000-132a | A05:5736612 | GGTAACCCTC | 8 | G | 2 | No |
| *Bna.CytP450.A5* | G2000-243-1 | A05:5736620 | TC | 12 | T | 2 | No |
| *Bna.CytP450.A5* | G2000-83b | A05:5736668 | CT | 0 | C | 13 | Yes |
| *Bna.FAD3.A5* | G2000-207b | A05:8104460 | C | 10 | CTGTGGATGATAGGGACGTGGCCTTTAAACTTTAAAGCATAAAACCGACTAACATCATCAGGAAAATAAAGTATCAAA | 3 | No |
| *Bna.FAD3.A5* | G1750-516a | A05:8104460 | CGT | 7 | C | 2 | No |
| *Bna.GTR1.A6a* | g1750-153-1 | A06:11068670 | TA | 0 | T | 10 | Yes |
| *Bna.GTR2.A6* | G1750-327a | A06:34290460 | GC | 3 | G | 4 | Yes |
| *Bna.FAE1.A8* | G2000-173b | A08:16404035 | GA | 3 | G | 2 | Yes |
| *Bna.CER4-like.A8* | G2000-270-2 | A08:16722892 | CCCT | 8 | C | 2 | No |
| *Bna.CER4-like.A8* | G1750-320a | A08:16723551 | GAA | 7 | G | 2 | No |
| *Bna.GTR2.A9a* | g1750-243-2 | A09:4452330 | AT | 12 | A | 2 | No |
| *Bna.GTR2.A9a* | G2000-103b | A09:4452349 | AC | 6 | A | 2 | No |
| *Bna.GTR2.A9a* | G2000-296-2 | A09:4453030 | CTGG | 8 | C | 2 | No |
| *Bna.GTR2.A9a* | G2000-155a | A09:4453031 | TG | 8 | T | 4 | No |
| *Bna.GTR2.A9a* | G2000-396-1 | A09:4453814 | TGA | 7 | T | 8 | Yes |
| *Bna.GTR2.A9a* | G2000-105b | A09:4453820 | TA | 6 | T | 2 | No |
| *Bna.GTR2.A9b* | G2000-297-2 | A09:4464042 | GA | 11 | G | 2 | No |
| *Bna.GTR2.A9b* | G1750-417a | A09:4464865 | A | 3 | AG | 2 | No |
| *Bna.GTR2.A9b* | G2000-302-1 | A09:4465416 | CTGG | 15 | C | 2 | No |
| *Bna.GTR2.A9b* | G2000-296-2 | A09:4465686 | TCGTACA | 14 | T | 4 | No |
| *Bna.CER4-like.C1* | G1750-437a | C01:2661762 | TG | 12 | T | 2 | No |
| *Bna.CER4-like.C1* | G2000-559a | C01:2661792 | TA | 9 | T | 2 | No |
| *Bna.VTE4.C2a* | g2000-403-1 | C02:15001410 | TA | 0 | T | 7 | Yes |
| *Bna.VTE4.C2a* | G2000-58b | C02:15002094 | TGCC | 17 | T | 5 | No |
| *Bna.VTE4.C2a* | G1750-489b | C02:15002099 | CCGT | 0 | C | 7 | Yes |
| *Bna.GTR2.C3* | G1750-318b | C03:41809049 | ATCATTGGAACACTATCAAACCTTTTGGTTTATTT | 14 | A | 2 | No |
| *Bna.GTR2.C3* | G2000-14a | C03:41809575 | CT | 7 | C | 2 | No |
| *Bna.GTR2.C3* | g2000-343-1 | C03:41810180 | C | 7 | CTCTGGA | 2 | No |
| *Bna.GTR1.C3a* | g1750-203-1 | C03:45116438 | TTG | 15 | T | 2 | No |
| *Bna.GTR1.C3b* | G2000-187a | C03:55172208 | TC | 3 | T | 2 | No |
| *Bna.FAE1.C3* | G2000-223a | C03:67615616 | GTT | 9 | G | 2 | No |
| *Bna.FAE1.C3* | G2000-550a | C03:67615804 | T | 6 | TTTAG | 3 | No |
| *Bna.FAE1.C3* | G2000-550a | C03:67615818 | CGA | 11 | C | 3 | No |
| *Bna.FAE1.C3* | G2000-550a | C03:67615841 | C | 11 | CG | 3 | No |
| *Bna.FAE1.C3* | G2000-550a | C03:67615891 | CGCA | 8 | C | 2 | No |
| *Bna.FAE1.C3* | G2000-23a | C03:67615898 | A | 19 | ATTTG | 4 | No |
| *Bna.FAE1.C3* | G1750-352a | C03:67615998 | GC | 6 | G | 2 | No |
| *Bna.FAD3.C3* | G2000-69b | C03:9721386 | CT | 9 | C | 2 | No |
| *Bna.CytP450.C4a* | g2000-457-1 | C04:11325698 | GGCGGA | 19 | G | 11 | No |
| *Bna.CytP450.C4a* | G2000-37a | C04:11325707 | C | 12 | CAG | 12 | No |
| *Bna.CytP450.C4a* | G2000-158b | C04:11326252 | C | 7 | CT | 2 | No |
| *Bna.CytP450.C4a* | G1750-347a | C04:11326284 | C | 18 | CG | 5 | No |
| *Bna.CytP450.C4a* | G2000-301-1 | C04:11326286 | C | 7 | CAG | 2 | No |
| *Bna.CytP450.C4a* | G1750-437a | C04:11326291 | A | 10 | AATC | 3 | No |
| *Bna.CytP450.C4a* | g1750-292-2 | C04:11326296 | TGGTCC | 10 | T | 2 | No |
| *Bna.CytP450.C4a* | G1750-347a | C04:11326336 | A | 14 | ACC | 3 | No |
| *Bna.FAD3.C4b* | G1750-318b | C04:56988424 | CT | 9 | C | 2 | No |
| *Bna.FAD2*.C5 | G1750-356a | C05:51453809 | T | 5 | A | 4 | Yes |
| *Bna.FAD2*.C5 | G2000-564a | C05:51453920 | C | 7 | T | 2 | No |
| *Bna.FAD2*.C5 | G2000-564a | C05:51453927 | T | 7 | C | 2 | No |
| *Bna.FAD2*.C5 | G1750-504a | C05:51454035 | T | 2 | C | 2 | No |
| *Bna.FAD2*.C5 | G2000-161a | C05:51454164 | C | 5 | A | 2 | No |
| *Bna.FAD2*.C5 | G2000-156b | C05:51454282 | G | 6 | T | 2 | No |
| *Bna.FAD2*.C5 | G2000-232b | C05:51454351 | C | 9 | A | 2 | No |
| *Bna.GTR2.C9* | g2000-408-2 | C09:5200267 | A | 10 | ACAC | 3 | Yes |
| *Bna.GTR2.C9* | G2000-302-1 | C09:5200666 | GGTTCT | 0 | G | 2 | No |
| *Bna.GTR2.C9* | G2000-295-1 | C09:5202504 | CGTA | 6 | C | 4 | No |
