# Supplementary material for: Genomics of predictive radiation mutagenesis in oilseed rape: modifying seed oil composition
Source: Plant Biotechnol J. 2023 Nov 3;22(3):738–50. doi: 10.1111/pbi.14220 (PMC10893948; doi:10.1111/pbi.14220)
Supplement: Supplementary file 8 — Table S2 Primers used in the validation of InDels. Oligonucleotide primers used for validation analyses. [file PBI-22-738-s007.docx]

Supplementary Information File 3. Primers used in the validation of InDels

| Target gene | Forward Primer | Sequence 5'-3' | Reverse Primer | Sequence 5'-3' |
| --- | --- | --- | --- | --- |
| CER4-like.A1 | CER4-like A1 F1 | GTTTGCGTAGTCCCATCTATAC | CER4-like A1 R1 | AACCCTGTGTTCTAACCTGATG |
| CER4-like.A1 | CER4-like A1 F2 | GTCGATGAGGGTAAGGCTATC | CER4-like A1 R2 | CAGGGAATGGTTCTTTGATAGTG |
| CER4-like.A8 | CER4-like.A8 F2 | GTTGTAGACCGATGGAAAAAGTC | CER4-like.A8 R2 | TCGCCTTAACCTAAGCTAAATG |
| CER4-like.A8 | CER4-like.A8 F3 | TAAGTAGTCCCAAATCCCAATACAG | CER4-like.A8 R3 | GGAATGGTTCTTTGAAAGTGCTAG |
| CER4-like.C1 | CER4-like.C1 F2 | GTTACTGTTGTCAGTGGGGACA | CER4-like.C1 R2 | GCGAAATTCAAGACATTGAGGACG |
| CytP450.A5 | CytP450.A5 F2 | GCCAAACAATGTCTTTTGAGAGGT | CytP450.A5 R2 | GTTGTTGTAGAGCAGTGTAGGTG |
| CytP450.A5 | CyP450.A5.FP | GTCCCGATGCCTTCCACCTCA | CyP450.A5.RP | GGACTCGTGCGGCCATATCCTTA |
| CytP450.C4a | CytP450.C4a F1 | CAACTACCTCGTCGGGAGATTC | CytP450.C4a R1 | CATGGATCCGAGGTTGAAGAGA |
| CytP450.C4a | CyP450.C4a.FP | GCTCTGCTAGACTCGGAGCCT | CyP450.C4a.RP | CCTTCCTGGTCTCGCTAAACCT |
| eIF(iso)4E.A4 | eIF(iso)4E.A4 F2 | GAGAACGTAGGTCTTTAAAACC | eIF(iso)4E.A4 R1 | TCTATCACTCATCAGCCTCATC |
| eIF(iso)4E.A4 | eIF(iso)4E.A4 F4 | CGAGCCTTTCGTCAGAATCG | eIF(iso)4E.A4 R4 | ACTTGTGTCTTCTCTTCAGACAG |
| FAD2.A5 | FAD2.A5 F3 | CGGCTACCACTAACTTCTACAG | FAD2.A5 R3 | CGGACAAATGAAAATGCAACATTTC |
| FAD2.A5 | FAD2.A5 F4 | GTAACGGATGAGTTCTATAACATAAC | FAD2.A5 R4 | CACACATGAAAACAGAGAATCAAGAC |
| FAD3.A3 | FAD3.A3 F1 | GTTCCTTTCAGTTACCAGAAAG | FAD3.A3 R2 | CAAGTAAGTGACAGCGTCCAG |
| FAD3.A4 | FAD3.A4 F3 | TTCAACTACTTGCTGGTCGATCA | FAD3.A4 R4 | CGAGATCACTCACGGCATCA |
| FAD3.A4 | FAD3.A4 F1 | TACCCCAAAGTCCATCAGACC | FAD3.A4 F2 | GTCTCTACACATCTTCTCCCTC |
| FAD3.A5 | FAD3.A5 F1 | CCACCGTTTAAGATCGGAGATATC | FAD3.A5 R1 | AAATCTTGAAGTCAGTGACGAC |
| FAD3.A5 | FAD3.A5 F3 | GTTTATCTATCGTTCCTCGTTGG | FAD3.A5 R3 | GAGAGAAAGCTAGATCACTCACC |
| FAD3.C3 | FAD3.C3 F1 | CATCACTAATTATACACCTAACA | FAD3.C3 R | AACATTATGGACCAGCAAGTAGTT |
| FAD3.C4b | FAD3.C4b F1 | CCTTTTTCCAGTTTGAGGCTAG | FAD3.C4b R1 | AGATAAACAAGAGTGGCCAACAC |
| FAE1.A8 | FAE1.A8.Fpcorr | ATGACGTCCGTTAACGTAAAGCTCC | FAE1.A8.RP | CTGACTTACCTGAATCAGAATCAATTTTG |
| FAE1.C3 | FAE1.C3 F2 | CTCAAGCATGTTTAATCCAACTCCT | FAE1-C3_R | TTAACAGAAGATCCTTAACCCC |
| GTR1.A1 | GTR1.A1 F6 | CAGATTCCGATAATTCCACTCTCTG | GTR1.A1 R1 | GCCGAAGTTGATAGTGCCACTA |
| GTR1.A1 | GTR1.A1 F4 | CGGACCAGTTTAGGTAAAACTCT | GTR1.A1 R5 | AGATCAGACATAGCTCTTGTCG |
| GTR1.A1 | GTR1.A1 F5 | TCTCTCTGTTGCTTTATGTTTCTG | GTR1.A1 R5 | AGATCAGACATAGCTCTTGTCG |
| GTR1.A6a | GTR1.A6a F2 | CTGGGAGTTACAGCAGTTAAAAG | GTR1.A6a R1 | CCCAACATCAGAAACAAAATCTGC |
| GTR1.C3a | GTR1.C3a F2 | GAATAATAAAGTGTCCAGTCCAA | GTR1.C3a R1 | GAGATGATCTGCGCGAAC |
| GTR1.C3b | GTR1.C3b F3 | CGACCAGTTCAGGTATACTC | GTR1.C3b R2 | GTGAATGTTCAGTTGTCCT |
| GTR2.A6 | GTR2.A6 F1 | GACAGGTCTAACAATGAAGAACAGA | GTR2.A6 R2 | CAAGAAAGCCGAGACCCAG |
| GTR2.A9a | GTR2.A9a F1 | ATTGGGAAGCTGCTCTTCGTG | GTR2.A9a R3 | CAGCCGTTATAACATGAGCTAC |
| GTR2.A9a | GTR2.A9a F2 | TCTTCGTGTGGTTCGGTTTCTC | GTR2.A9a R4 | GGCTGTTTCACTGACTTTAACCT |
| GTR2.A9b | GTR2.A9bF1 | GACTGTCTCCACCATCTTCT | GTR2.A9bR1 | CTGTGCCAGCAAAGGCAG |
| GTR2.A9b | GTR2.A9b F3 | ACTGCTGCAGTCCCACAG | GTR2.A9b R3 | GTTCCACTGGCTCTAACCTG |
| GTR2.A9b | GTR2.A9b F6 | CTTCATCGATCTACTACTTAACCATG | GTR2.A9b R6 | CCAAACGCGTCGGCTATTC |
| GTR2.C3 | FGTR2.C3 | CAACTGTATGATTCATGCAAAGC | R3GTR2.C3 | GACATTAGACTGGATATAGACGATC |
| GTR2.C3 | GTR2.C3 F4 | GTTTCTTGTAGTTGGAGCAGGC | GTR2.C3 R5 | CTTCTTAAGGTAGGCACGAGG |
| GTR2.C3 | GTR2.C3 F5 | GCCAGATAGCGTTTCTACTGT | GTR2.C3 R6 | CCTGTCATCAAAAAGACAACGTAG |
| GTR2.C9 | GTR2.C9F | TGATCGTCGCTTAGGCTCT | GTR2.C9R | CTCTTCATCATCACTTCCTTTGTACT |
| GTR2.C9 | GTR2.C9 F3 | CACTAACACATGTCTCTGTCAC | GTR2.C9 R4 | TAATCCCTCCTGCTCCCACC |
| VTE4.A2a | VTE4.A2a F2 | TCTTGTTCCGCCAACCTCTC | VTE4.A2a R2 | CTTGAGCTTCCGCCGATC |
| VTE4.A2b | VTE4.A2b F2 | CCAGGCACAAAGTATCTTCC | VTE4.A2b R1 | CAAGGGATAAACAGAAAGGCTAT |
| VTE4.C2a | VTE4.C2aF1 | AATTTTTAGATATTGAAAGCAGG | VTE4.C2aR2 | TTAGATTCTGTTTTCTAACAGGTACTG |
